# Regulation of Prostitution—Results of the Attempt in St. Louis, Missouri

**Published:** 1871-05

**Authors:** Edmund Andrews

**Affiliations:** Professor of Principles and Practice of Surgery in Chicago Medical College


### REGULATION OF PROSTITUTION—RESULTS OF THE ATTEMPT IN ST. LOUIS, MISSOURI.

By EDMUND ANDREWS, M.D., Professor of Principles and Practice of Surgery in Chicago Medical College.

The recent attempt to register, license, and regulate the "Social Evil" in St. Louis appears to be making the same disastrous failure which has attended similar attempts in Europe. A committee of the common council reports already the police are confronted by their total inability to maintain the registry.

When the plan was first put in operation it appears to have been favored by the prostitutes, and the police registered 718 names, to which they soon added 228 more, making 946 registered women in all. Now, the names have fallen off to the extent of 467. In other words, the police have lost almost exactly half the women from their control, even in the short time that has already elapsed. Similar results have occurred, according to Mr. Berkely Hill, under the "Contagious Diseases Act," in certain parts of England, (see *British Medical Journal*). There, the registered women have fallen from 1960 to 565, or, in other words, the police have lost nearly three-quarters of the women from their control.

The Continental experience is the same, the testimony of the superintendents of the police being that the women evade and defy the registry, and in annually increasing numbers hasten to go over to the class of unregistered clandestines.

The *New York Medical Gazette* thinks that the diminution of prostitutes is not due to the women going into clandestine vice, but to a positive improvement of public virtue produced by the law. The opinion of the Continental police, however, is different, as already stated, and indeed the plainest common sense supports their view.

If the regulation law of St. Louis, while affording increased facilities and attractions to licentiousness, has already reformed half the strumpets of that city, and the "Contagious Diseases Act" three-quarters of those of the garrison towns of England, they must be more easily purified than those of Chicago. I, for one, have no faith that half the fast men and loose women, either of St. Louis or England, have suddenly reformed in consequence of a law being passed allowing them to practice prostitution with impunity. The statistics look rather too rosy to be true. It is to be suspected that the frail women, like their sisters in Germany and France, found the police surveillance and registry more unprofitable, or less agreeable than they expected, and withdrew into clandestine vice.

A similar suspicion rests on the report of diminished disease in the brothels and public institutions. The number of diseased women is given at 40 less than when the law went into operation, while the venereal cases in public institutions are reported as diminished about 75 per cent. Are we to understand that these 40 women were the authoresses of three-quarters of all the venereal disease in the hospitals of St. Louis? Certainly there is something out of joint in this calculation which needs further investigation.

